# A randomised controlled trial of feedback to improve patient satisfaction and consultation skills in medical students

**DOI:** 10.1186/s12909-020-02171-9

**Published:** 2020-08-20

**Authors:** Michelle M. Y. Lai, Noel Roberts, Mohammadreza Mohebbi, Jenepher Martin

**Affiliations:** 1 Medical Student Programs, Eastern Health Clinical School, Monash University and Deakin University, Level 2, Arnold Street, Box Hill, VIC 3128 Australia; 2 Curtin University Medical School, Perth, Australia; 3 Biostatistics Unit, Faculty of Health, Deakin University, Geelong, Australia

**Keywords:** Medical students, Clinical competence, Clinical skills, Medical education, Formative feedback, Patient satisfaction, Health behaviour, Randomised controlled trial

## Abstract

**Background:**

The use of feedback has been integral to medical student learning, but rigorous evidence to evaluate its education effect is limited, especially in the role of patient feedback in clinical teaching and practice improvement. The aim of the Patient Teaching Associate (PTA) Feedback Study was to evaluate whether additional written consumer feedback on patient satisfaction improved consultation skills among medical students and whether multisource feedback (MSF) improved student performance.

**Methods:**

In this single site, double-blinded randomised controlled trial, 71 eligible medical students from two universities in their first clinical year were allocated to intervention or control and followed up for one semester. They participated in five simulated student-led consultations in a teaching clinic with patient volunteers living with chronic illness. Students in the intervention group received additional written feedback on patient satisfaction combined with guided self-reflection. The control group received usual immediate formative multisource feedback from tutors, patients and peers. Student characteristics, baseline patient-rated satisfaction scores and tutor-rated consultation skills were measured.

**Results:**

Follow-up assessments were complete in 70 students attending the MSF program. At the final consultation episodes, both groups improved patient-rated rapport (*P* = 0.002), tutor-rated patient-centeredness and tutor-rated overall consultation skills (*P* = 0.01). The intervention group showed significantly better tutor-rated patient-centeredness (*P* = 0.003) comparing with the control group. Distress relief, communication comfort, rapport reported by patients and tutor-rated clinical skills did not differ significantly between the two groups.

**Conclusions:**

The innovative multisource feedback program effectively improved consultation skills in medical students. Structured written consumer feedback combined with guided student reflection further improved patient-centred practice and effectively enhanced the benefit of an MSF model. This strategy might provide a valuable adjunct to communication skills education for medical students.

**Trial registration:**

Australian New Zealand Clinical Trials Registry Number ACTRN12613001055796.

## Background

Multisource feedback (MSF) is widely used as a quality improvement strategy in healthcare, based on the assumption that performance feedback from more than one source, such as self, colleagues or patients would prompt healthcare professionals to modify their clinical practice [1–5]. Leading medical regulators, including Medical Councils in the UK and Australia, and College professional bodies have incorporated MSF into the performance review cycle for doctors [5]. Although MSF is a proven feasible, valid and reliable method to assess key competencies such as communication skills among doctors in practice [3, 4], the users of patient feedback cite a weak evidence base [6–9]. In systematic reviews of trials using feedback as core interventions, concerns persisted in the variable effectiveness of feedback [5, 10].

Although MSF has shown small and potentially important improvements in clinical practice and professionalism in doctors [5, 11], the strategy has not been routinely integrated into education for medical students, and rigorous evidence from randomised trials to support its use is currently lacking [12–14]. In the context of growing populations of people living with chronic complex conditions, and improved health literacy, it is a priority to help medical students learn about professionalism and communication skills. Such skills have been shown to enhance patient outcomes in symptom relief, adherence to treatment, and patient satisfaction and are recognised core competencies in physicians [15, 16]. However, genuinely useful clinical education through feedback is challenging, and the best way to deliver it remains unclear. Traditional feedback models in healthcare education may vary in structure but are usually reductionist in approach and educator driven, which may hinder effective delivery. Instead, feedback strategies that incorporate reflection-in-action in a supported sequential learning process are considered highly desirable [11].

Using these strategies, we implemented a teaching program to enhance clinical skills and professionalism among junior clinical medical students in Victoria, Australia. Patient Teaching Associates (PTA), who are ambulatory patient volunteers living with chronic conditions, describe their personal story in a simulation clinic [17]. In addition to oral MSF during the simulation episode, students in the intervention group of this study also receive written structured patient feedback based on patient satisfaction scores.

In the current study, we examined whether additional written patient feedback to medical students after ambulatory consultations improved the medical students’ clinical consultation performance in this program and hypothesised that combining a range of modalities of feedback would improve student consultation. The primary objective of this study was to identify whether additional written feedback from patients to students improved patient satisfaction and tutor reported student consultation skills. The secondary objective was to examine whether the MSF model improved patient satisfaction and tutor reported student consultation skills over one semester.

## Methods

### Study oversight, settings and participants

The trial (Australian New Zealand Clinical Trials Registry Number 12613001055796) was conducted from March 2013 to March 2014 at Monash University Eastern Health Clinical School (EHCS), Victoria, Australia and was supported by a local educational grant from the University. The protocol of this trial has been published elsewhere [18]. The statistical analysis was supervised by a statistician from Deakin University, who performed analysis using the de-identified database and was not involved in student assessment.

The setting of the teaching program was an after-hours general practitioner clinic that was adapted as a medical student teaching clinic during the day. The Patient Teaching Associate (PTA) program recruited real patients with chronic illnesses from the community and aimed to promote a patient-centered approach to the consultation [19]. Our PTAs have a wide range of chronic diseases such as diabetes, musculoskeletal conditions, renal disease, respiratory disease, cancer of various types, Parkinson’s disease. Many have comorbidities. The most common problems were diabetes, musculoskeletal and chronic obstructive pulmonary disease. Clinical tutors were senior medical practitioners, including general practitioners, physicians and surgeons. All tutors received instructions, a protocol and the tutor assessment and feedback framework relating to the 20-item Rating Instrument of Clinical Consulting Skills (RICS-20) [19].

All third-year medical students in their first clinical year in 2013, enrolled in either Monash University or Deakin University, attending Eastern Health Clinical School, and willing to participate in the teaching program were eligible for inclusion in this study. Medical students led a general practitioner style consultation and reviewed the same patients in groups of three students. There were no exclusion criteria. All participants provided written consent.

The study was conducted with no deviation from the published planned procedures. Briefly, after enrolment, student participants completed a common assessment battery, including demographics, baseline patient satisfaction scores and tutor assessment scores at the baseline assessment before randomisation. A group of three students saw the same patient and received individual assessments from the same tutor and the patient in each station during a consultation episode. The same student group rotated to a different station in the next consultation episode.

### Interventions

All students participating in the PTA program in both the intervention and control group attended a one-hour briefing meeting. All supporting materials were provided to students in digital form. All students in both intervention and control groups received immediate oral formative feedback from the tutor, patient volunteers and peer students (MSF) towards the end of each student consultation episode and written tutor feedback according to the RICS-20 framework [20] returned to the student in the following week.

The educational intervention was the feedback of the completed 21-item Medical Interview Satisfaction Scale (MISS-21) in addition to usual oral feedback [21]. The intervention pack included patient feedback questionnaires for all previous consultations by the student as well as written instructions about self-reflection on the feedback received based on the Pendleton feedback framework [22]. MISS-21 is a widely available 21-item validated visit-based tool questionnaire for measuring patient satisfaction in the primary care context. The MISS-21 questionnaire was published in appendix 1 of the article by Meakin et al. [21]. Students in the intervention group received their intervention pack no later than 1 week before the last consultation during one semester (generally six consultations in total).

Following the distribution of intervention packs, email adherence reminders were sent to emphasise the importance of following study guidelines to read the written feedback. A final student consultation episode was scheduled within the last 2 weeks of the same semester. Adverse effects of feedback were monitored as per study protocol [18]. All students were asked to contact the program coordinator separately to this research study if they experience problems related to any feedback in the education program. Debriefing and referral for counselling pathways were available.

### Measures

Student performances were measured using patient assessment scores (MISS-21) and tutor assessment scores (RICS) to assess the effects of 1. written feedback and 2. the overall effect of multisource feedback.

#### Patient satisfaction scores (MISS-21)

The primary outcome measure was patient satisfaction scores obtained within the same day after the student consultation episodes using MISS-21 [21]. The consultation satisfaction questionnaire has been validated to rate general practitioners and nurse practitioners for feedback and educational purposes. It consists of 21 individual consultation-based statements with subscales referring to distress relief, communication comfort, rapport and compliance intent. Its internal consistency measures have been reported [21]. Patient volunteers were asked to indicate their level of agreement with the statements on a 7-point Likert scale. The instrument was chosen because of its ease of administration, as it is visit based, free from cost or facility-based questions and is a valid and reliable feedback tool in the consultation-based clinical setting [23–25]. All PTAs were orientated to the MISS-21 instrument for clarification of the items and terminology. An independent co-worker interviewed each PTA after each consultation to obtain the scores. As this study recruited junior clinical students in their first clinical year, we did not request a mandatory full completion of MISS-21 and provided an option of ‘not applicable’ in the sub-scale of compliance intent which was not always relevant in this context, for instance, if the consultation episode did not involve student advice on medical management.

#### Tutor-rated clinical skills (RICS-20)

Secondary outcomes included the tutor rated RICS-20 with a composite performance score in consultation skills and four subscale scores of patient-centred approach, history taking, physical examination and problem-solving and management. Tutors assessed student behaviour and clinical skills by observing the encounter between students and patients and were required to complete the assessments within 24 h after the student consultation episodes. All tutors received standardisation training based on a video. Tutor assessors were asked to appraise consultation performance using the medical intern level as a benchmark. RICS-20 is a student performance assessment tool designed for the Patient Partnership Program (P^3^), a teaching program developed at the Launceston Clinical School, University of Tasmania [26, 27] on which the PTA program translated. Its construct validity and psychometric properties have been reported [28]. The concurrent use of RICS-20 avoided the risk of a simple training effect on MISS-21 scores.

### Sample size

In the power calculation, we used an unpaired t-test to detect a post-intervention difference in the primary outcome (MISS-21) between the two groups. We incorporated the standard deviation used in the nurse practitioner group in a trial using MISS-21 measurement [27]. With 33 participants per group, there was 80% power of detecting a difference of at least 0.32 points in the MISS-21 at 5% significance level, assuming the standard deviation in the control group is 0.46 [29].

### Randomisation and allocation procedure

Assignment of interventions was performed by block randomisation, according to a list of computer-generated random numbers. Allocation numbers were kept in sealed containers. Tutors, patient assessors and data analysts were blinded to group membership. Because of the nature of the feedback, student participants were not blinded to the group membership.

An investigator (ML) generated the allocation sequence using computer-generated numbers and concealed the random sequence in sealed opaque envelopes. Another investigator (NR) not directly involved in the assessment of students drew the envelopes and assigned participants to their study groups.

### Statistical methods

We compared student characteristics at baseline between the two groups using chi-squared test for categorical variables and t-test for continuous variables. An intention to treat analysis was performed using a linear mixed model approach. To assess the impact of the intervention on primary and secondary outcomes, time by intervention interactions were examined in a linear model that contained fixed effect intervention group allocation, fixed effect measurement time and time by intervention interaction. A two-level random effect model was implemented to take account of the student consultation group clustering effect (students consulted in randomly assigned groups of three that varied in follow-up consultations) and within-individual autocorrelation due to repeated measures for each participant. The overall *p*-values for time by intervention interaction and overall follow-up (subsequent consultation episodes) in both groups combined were reported. Time by intervention interaction impact and combined follow-up impacts and their 95% confidence intervals (CIs) were reported. Age, gender, education in years, postgraduate status and International student status may influence the proficiency in communication skills and/or commands in English language, and planned analyses of these baseline characteristics were determined a priori. All data were analysed using Stata 14 (StataCorp LP, College Station TX, USA). *P*-values of < 0.05 were considered significant.

*Cohen’s d* was used to examine the magnitude of such differences: Effect size of *d* 0.2 was considered as a small effect; a *d* of 0.5 as moderate effect and a *d* of 0.8 as a large effect.

#### Patient satisfaction scores (MISS-21)

All inter-item correlations were above the recommended 0.3 suggesting that the items within each subscale correlated well with the other items in that subscale. The overall Cronbach’s alpha was 0.93 (Cronbach’s alpha if item deleted range: 0.924–0.940) suggesting very good internal consistency of the scale [30]. Subscales Cronbach’s alpha were 0.95 for ‘Distress Relief’, 0.61 for ‘Communication Comfort’, 0.92 for ‘Compliance Intent’ and 0.91 for ‘Rapport’ indicating an excellent level of reliability except for ‘Communication Comfort’ subscale. While Cronbach’s alpha for ‘Communication Comfort’ subscale was questionable this subscale was used for further data analysis because inter-item correlations for the sub-scale were above the recommended 0.3, the overall Cronbach’s alpha showing excellent internal consistency and the fact that Cronbach’s alpha if item deleted index did not suggest any item deletion. In addition, retaining this subscale helps consistency and comparability of our findings with similar reports.

#### Tutor-rated clinical skills (RICS-20)

All inter-item correlations were above the recommended 0.3 indicating high agreement between items and subscales [30]. The overall Cronbach’s alpha was 0.98 (Cronbach’s alpha if item deleted range: 0.978–0.983) illustrating excellent internal consistency. Sub-scales Cronbach’s alpha was 0.92 for approach, 0.95 for management, 0.94 for clinical and 0.95 for history indicating an excellent level of reliability.

## Results

### Participant flow

**Fig. 1:**
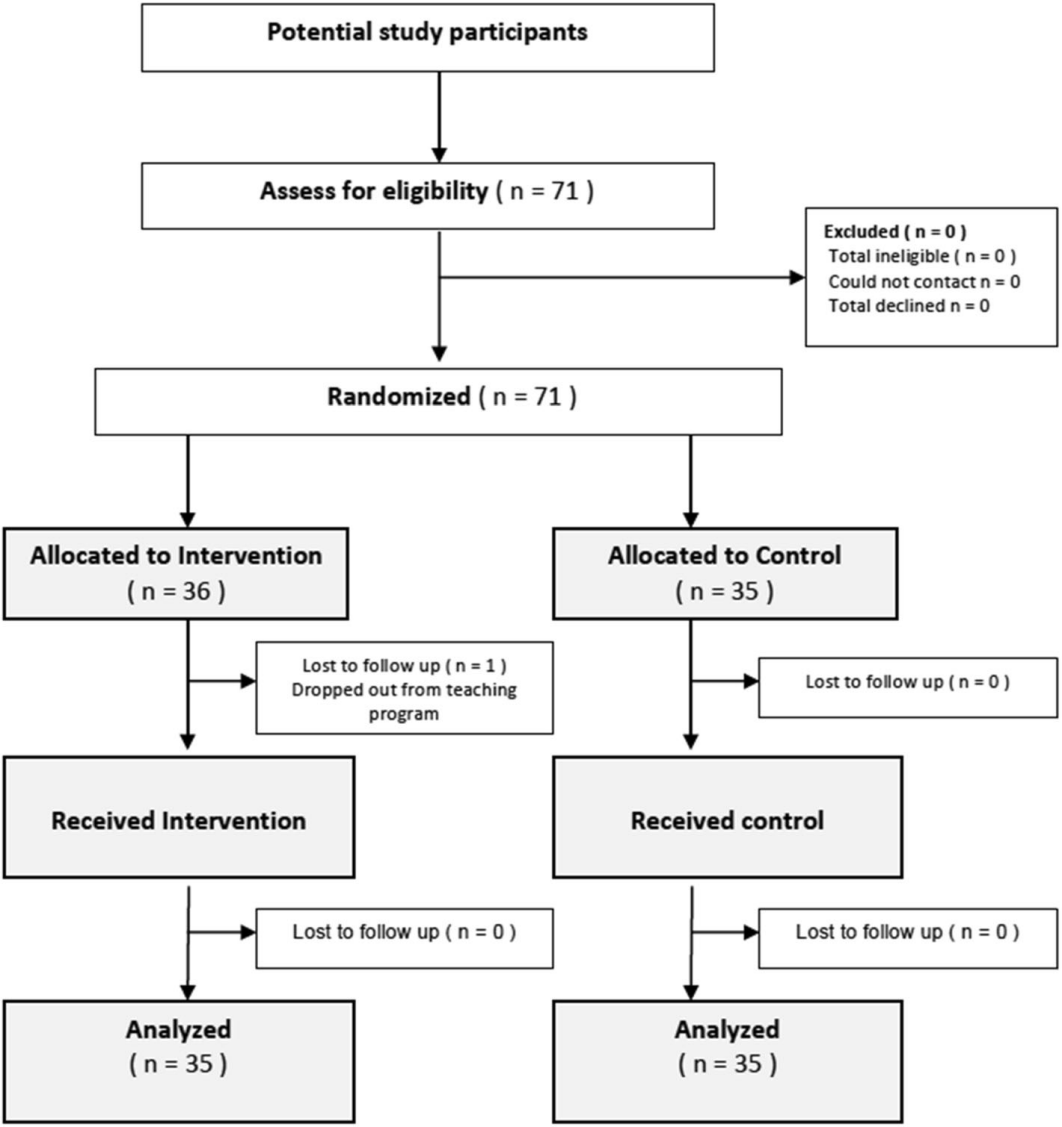
Trial profile of the PTA Feedback study. PTA = patient teaching associate, n = number

Figure 1 presents the flow diagram of participants in the PTA Feedback study. Of the 71 eligible medical students, all 71 participants were enrolled during the period between May 2013 to September 2013 and were allocated to the intervention group (*n* = 36) or the control group (*n* = 35). In the intervention group, one medical student completed the initial pre-assessment but dropped out of the teaching program and did not participate in the intervention and follow-up assessments. All the other 70 students completed baseline and follow-up assessments over one semester. The rate of adherence to the study protocol in the 70 students was 100%, including completion of a self-reflection exercise based on the feedback received. All students returned the form with a statement that they have read the written feedback and provided one’s own action plan following self-reflection on the feedback received in the intervention packs.

The analysis was carried out in 70 students according to ‘intention-to-treat’, meaning that analysis was performed on the data for the allocated groups, regardless of the individual’s level of participation in the program. Twenty-four patient teaching associates living with chronic diseases participated in this study. These patients are real patients who present themselves in a simulated consultation setting. Students did not see the same patient twice.

Table 1 shows the baseline characteristics of the two study groups. Covariates were balanced in the two groups after randomization. Hence, no adjustments for the variables were undertaken.

**Table 1:** Baseline characteristics of the medical students, Patient Teaching Associate Feedback Study

Student Characteristics	Patient feedback and usual multisource feedback (intervention) (***n*** = 35)	Usual multisource feedback (Control) (***n*** = 35)	All students (***n*** = 70)
Age at entry in years, *mean ± SD*	22.8 ± 3.7	23.4 ± 3.7	23.1 ± 3.7
Male	44.4%	51.4%	34 (47.9%)
Married^a^	2.9%	5.7%	3 (4.4%)
Single^a^	91.2%	88.6%	62 (89.9%)
Education in years, *mean ± SD*	16.0 ± 2.1	16.5 ± 2.5	16.2 ± 2.3
Completed undergraduate degree	38.9%	48.6%	31 (43.7%)
International student	11.1%	17.1%	10 (14.1%)
Language spoken at home			
English	77.8%	82.9%	57 (80.3%)
Not English	22.2%	17.1%	14 (19.7%)
Prior work experience			
Paid work	91.4%	86.1%	63 (88.7%)
Years of paid work, *mean ± SD*	4.2 ± 3.5	4.2 ± 3.6	4.2 ± 3.5
Volunteer work	41.7%	45.7%	31 (43.7%)
Years of volunteer work, *mean ± SD*	0.9 ± 1.6	1.4 ± 2.2	1.1 ± 2.0

^a^Marital status was not available for two students

#### Effect of written feedback

The overall patient-rated satisfaction scores using MISS-21 and its domains in distress relief, communication comfort and rapport did not differ significantly between the intervention and the control groups (Cohen’s d = 0.20). With regards to the tutor-rated clinical skills using RICS scores, patient-centeredness sub-scores for students in the intervention group improved by 0.74, from 3.22 (SD 0.81) to 3.96 (SD 0.85), showing significant difference from the control group (*P* < 0.005), which improved by 0.48, from 3.51 (SD 0.74) to 3.99 (SD 0.73). Sub-scores for clinical skills in history taking, examination as well as problem-solving and management did not differ significantly between the two groups (Table 2).

**Table 2:** Assessment outcomes of study participants, Patient Teaching Associate Feedback Study

Assessment scores	Intervention (***n*** = 35)		Control (***n*** = 35)		All students (***n*** = 70)		Between groups		
	Baseline score	Final score	Baseline score	Final score	Change from baseline	P	Interaction effect	P	Cohen’s d
**Patient rated MISS-21**^a^									
Distress relief	5.08	5.19	5.01	4.93	0.08 (0.4–0.55)	0.73	−0.18 (−0.49, 0.85)	0.60	−0.10
Communication comfort	5.78	5.91	5.55	5.93	0.39 (−0.07,0.85)	0.10	−0.25 (− 0.91,0.4)	0.44	− 0.18
Rapport	5.99	6.14	5.74	6.2	0.47 (0.17,0.77)	< 0.01*	−0.31 (− 0.73,0.11)	0.15	− 0.34
Compliance intent	5.41	5.59	5.07	6.16	1.10 (0.49,1.7)	< 0.01*	−0.92 (−1.71,-0.13)	0.02*	−0.73
Overall	5.64	5.77	5.49	5.76	0.27 (0.07,0.61)	0.12	−0.14 (− 0.62,0.34)	0.56	− 0.16
**Tutor rated RICS-20**^b^									
History taking	3.21	3.43	3.22	3.45	0.15 (−0.26,0.56)	0.47	−0.01 (− 0.59,0.57	0.97	− 0.09
Examination	2.61	3.86	3.07	3.39	0.22 (−0.25,0.68)	0.36	0.58 (−0.07,1.23)	0.08^	0.16
Problem solving & management	2.74	3.49	3.07	3.27	0.19 (−0.34,0.72)	0.48	0.56 (−0.19,1.3)	0.14	0.35
Patient centredness	3.22	3.96	3.51	3.99	0.26 (0.03,0.49)	0.03*	0.48 (0.16, 0.79)	< 0.01*	0.26
Overall	2.96	3.68	3.17	3.78	0.32 (0.06,0.59)	0.02*	0.28 (−0.09,0.64)	0.14	0.12

MISS-21, 21-item Medical Interview Satisfaction Scale (18); RICS-20, 20-item Rating Instrument of Clinical Consulting Skills (19); *CI* Confidence Interval *P < 0.05, ^*P* < 0.1. ^a^Random-effects variance 0.70 (within individuals), and 0.26 (consultation group); ^b^Random-effects variance 0.60 (within individuals), and 0.55 (consultation group)

#### Effects of MSF over time

**Fig. 2:**
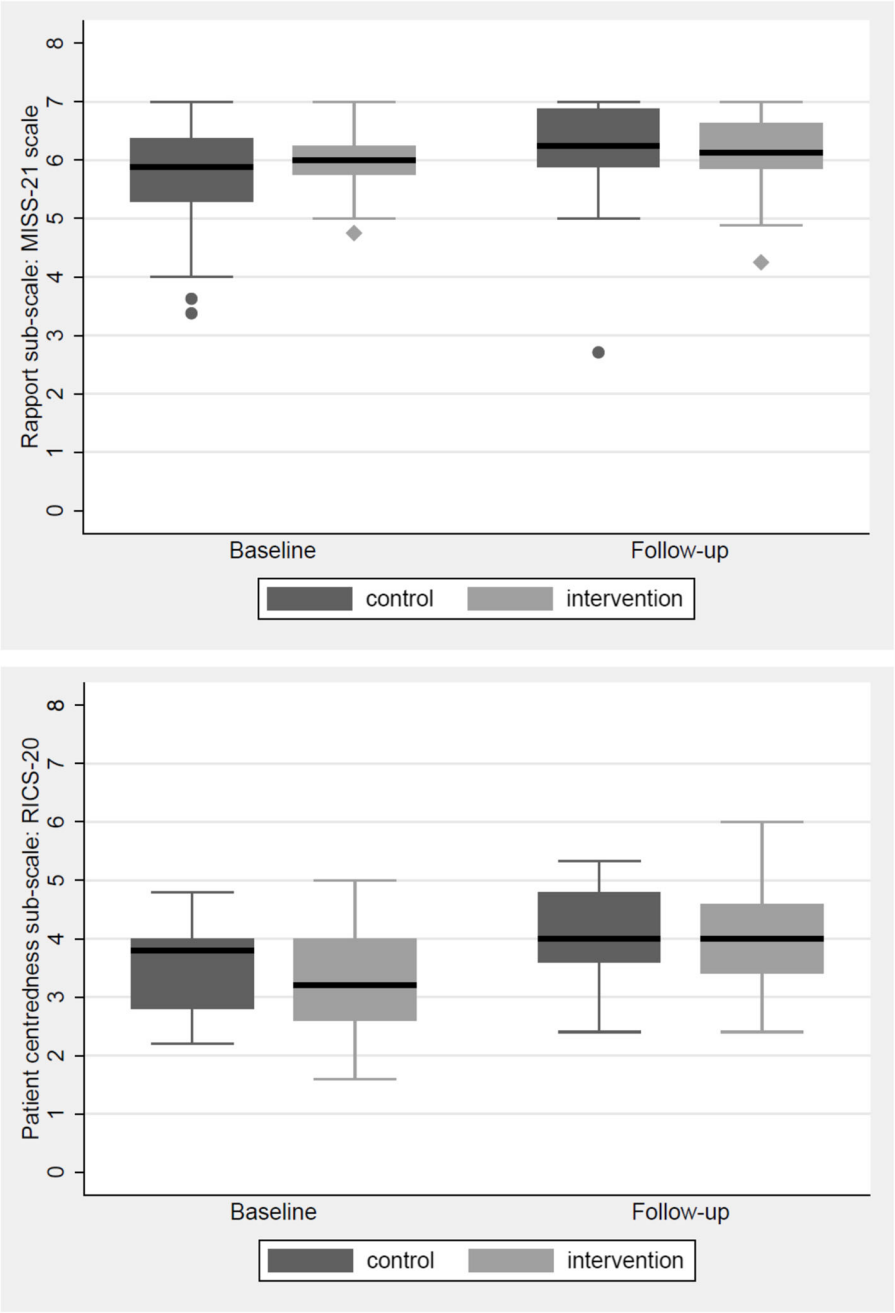
Boxplots showing the change in rapport and patient-centeredness scores over the study period. **a**. Rapport increased in both groups after the multisource feedback (MSF) program and **b**. patient-centeredness has been increased by patient satisfaction feedback intervention

For all students, rapport sub-scores of MISS-21 improved from 5.86 (SD 0.75) to 6.17 (SD 0.73) (*P* = 0.002) (Fig. 2a) and compliance intent sub-scores improved from 5.25 (SD 1.17) to 5.87 (SD 1.02) (*P* < 0.001) (Table 2). The overall MISS-21 mean scores showed improvement trend from 5.56 (SD 0.74) to 5.77 (SD 0.87) although *p*-value did not reach statistical significance (*P* = 0.12).

All students significantly improved overall consultation skills sub-scores from 3.06 (SD 0.73) to 3.73 (SD 0.81) (*P* < 0.05). The mean patient-centeredness sub-scores improved from 3.36 (SD 0.79) to 3.98 (SD 0.79) (*P* < 0.05) (Fig. 2b). History taking, examination and problem-solving sub-scores did not show significant improvements.

Pre-planned secondary analysis adjusting for age, gender, education in years, postgraduate status, and international student status has been performed on significant main outcomes. None of the covariates was significant, and no further adjustments were needed.

A priori decision was made to follow the natural hierarchy of data in the main and secondary outcome analyses. Hence no alternative random effect models were tested. Within individual variance component was relatively large and the student consultation group had a moderate impact (refer to Table 2).

### Adverse effects

Unblinding was carried out in one participant for debriefing purpose, before receiving the intervention, as the student dropped out of the teaching program. No students reported distress from reading written feedback or performing self-reflection task using the intervention pack.

## Discussion

These findings add to the evidence that MSF significantly leads to practice improvement. This randomised trial showed that, in both arms of the study, clinical medical students’ behaviour was modified and showed measurable impact in their consultation performance after exposure to the MSF teaching model. Specifically, patient-rated rapport and compliance intent, as well as tutor-rated patient-centeredness and overall consultation skills, significantly improved in following repeated use of MSF after one semester. These domains are highly essential core competencies in Patient-Centred Care (PCC) that have been shown to impact on patient outcomes among practising clinicians [15, 16]. Individualised written patient satisfaction feedback in the intervention group further significantly improved tutor-rated patient-centeredness as compared with the control group. These findings suggest that structured, written consumer feedback on patient satisfaction with guided self-reflection effectively enhanced the MSF model in medical student education. This finding of providing patient satisfaction feedback to medical students that improved patient outcome is consistent with observations in an earlier pre-post study in physicians who were given real-time patient satisfaction score feedback (combined with education and incentives as intervention) [8].

Our study design consists of elements that are considered highly desirable in a Cochrane study that reviewed the effect of feedback on professional practice: a study population with a low performance baseline, feedback provided more than once and directed towards an action plan using a facilitative framework in promoting critical reflection on the written feedback [10]. The teaching clinic setting for students in their first clinical year was uniquely designed for repeated practice of consultation skills and a combination of feedback delivered in both verbal (usual mode) and written formats in the intervention group, better accommodating a range of preferred learning styles [31]. To promote effective learning from individualised written patient feedback, and to further modify students’ behaviour for learning, we provided a facilitative framework to guide students in their critical reflection on the written feedback. Students showed a high compliance rate in formulating their own plan for performance improvement.

Researchers have reported concerns about the variable methods to deliver feedback in different training models affecting the effectiveness of feedback in improving practice [5, 10], and the negative effect of non-specific or lengthy feedback which could be viewed as frustrating and unhelpful [32]. In our program that incorporated patient feedback to medical students, patients may find it challenging to articulate concepts of PCC when asked to provide oral textual feedback during the teaching episode. However, use of the additional written feedback tool shortly after the consultation may help guide patients in scoring various dimensions of PCC and provide a useful adjunct to motivate self-reflection among the medical students.

MISS-21 is a tool to facilitate focused feedback on consumer satisfaction and measured subcomponents in PCC that are priorities in student learning: distress relief, communication comfort, rapport and improving compliance [21]. Hence, not only the structured assessment tool served to remind students of the intended learning outcomes, but it also shaped the feedback of patients to capture specific dimensions of PCC and reduce variability in the learning experience related to the use of real patients. Using a tool to relate individuals’ performance to the success criteria can signal a gap in the level of performance and desired goal in learning. Studies have suggested that resolving this gap could motivate higher levels of effort [32, 33].

The participation of real patients was an important enabler to the success of this program. Real patients are increasingly fulfilling active teaching roles in developing medical students’ communication skills and understanding of factors affecting health and health care [34, 35]. Recruiting ambulatory patient volunteers decreases relying on hospitalised patients who are often unwell [36], which is a real challenge facing medical educators today. In contrast to simulated patients, real patient teachers share their unique insights based on their experiences in real social contexts with genuine conditions impacting on daily life [35]. Besides enhancing the integration of technical and interpersonal skills, their effectiveness in teaching physical examination techniques could also be comparable to physicians, in terms of OSCE results [37]. Real patients could highlight the patients' perspective and give feedback on subjective aspects of the physical examination [35, 37]. This capacity might explain our findings that students in the intervention group receiving additional patient feedback tended to show better tutor-rated physical examination scores (*P* = 0.08), compared with the control group.

### Strengths and limitations

We consider that the findings provide important evidence and information to enhance our approach in applying MSF to improve specific dimensions of PCC. The strengths of this trial include an experimental study design that provides more robust research methodology than observational studies, such as before and after comparisons, and the use of validated and reliable structured feedback scales. The program in this trial covered a wide range of conditions in people of various ages rather than limiting the student consultations to specific groups. We have recruited a group of highly motivated students and patient volunteers leading to a very high adherence to the study protocol and low drop-out rate. The study was not affected by the poor response rate often found with postal surveys in population-based studies.

Larger studies are recommended. While this study had sufficient statistical power to detect moderate intervention impacts, some of the observed effects were small.

Our results are generalisable to medical students learning management of ambulatory patients living with chronic conditions in Australia, but not to inpatient settings or non-medical health students and practising doctors, who have a higher performance baseline. We have not included multicultural patients, and our outcome measures did not cover all aspects of PCC, patient health status or quality of life outcomes, because patients were instructed not to follow the students’ management plans arising in the simulated practice environment. This study did not examine PCC from the viewpoint of family and carers who attended the consultation episode. Furthermore, other factors could have contributed to improved consultation skills during the study period. The study design did not limit students from clinical practice with other patients in the hospital. The intervention pack included the patients’ written feedback and the reflection process as part of the education intervention. Further studies are needed to identify if the communication mode, the reflection or the combined methods would improve the student performance.

## Conclusions

These findings have shown that the use of MSF from tutor, peers, self and importantly, the patient as consumer, is an effective and highly feasible strategy in medical student education and specifically in learning non-technical skills. The use of the multimodality approach can provide both depth in qualitative and breath in quantitative feedback. Structured written feedback of patient satisfaction combined with guided student reflection, effectively enhanced the benefit of an MSF model and provided a valuable adjunct to communication skills education in medical students.

## Supplementary information

**Additional file 1.**

## Data Availability

The datasets used during the current study are available from the Monash University, but restrictions apply to the availability of these data and are not publicly available. Data are however available from the corresponding author upon reasonable request and with permission of Monash University.

## Abbreviations

MISS-21: The 21-item Medical Interview Satisfaction Scale
MSF: Multisource feedback
P^3^: Patient Partnership Program
PCC: Patient Centred Care
PTA: Patient Teaching Associates
RICS-20: The 20-item Rating Instrument of Clinical Consulting Skills
