# Supplementary material for: A randomised controlled trial of feedback to improve patient satisfaction and consultation skills in medical students
Source: BMC Med Educ. 2020 Aug 20;20:277. doi: 10.1186/s12909-020-02171-9 (PMC7439652; doi:10.1186/s12909-020-02171-9)
Supplement: Supplementary file 1 — Additional file 1. [file 12909_2020_2171_MOESM1_ESM.docx]

Appendix 1:

A guided self-reflection exercise worksheet

Affix Label for participant ID


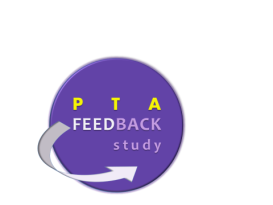
**Worksheet for feedback on patient satisfaction**

**Date:**

We hope you can gain the most out of the real patient learning experience in the PTA Program. Please complete the exercise below and return your completed worksheet at your final PTA session to Mr Noel Roberts, PTA program manager.

**What do I receive?** The forms in this envelope are the written feedback of patient satisfaction to you alone (not your group) using MISS-21. The patient volunteers completed them for you immediately after your consultation sessions with them in the past weeks.

**What is MISS-21?** The MISS-21 is a validated audit tool and is popular among GP for quality improvement activities and can be applied to one single visit. Using the questionnaire, your patient volunteers provided feedback from their perspectives in the following domains: distress relief, communication comfort, rapport and compliant intent if applicable. These are also suggestive of the level of patient centeredness of your clinical consultations. While we use it as the most appropriate validated audit tool available, it is recognised that depending on your role with the specific consults, some of the statements may not apply or may be difficult for the patient to rate.

**How to use the MISS-21 feedback?** To benefit from the feedback, we invite you to spare 10 minutes for a self-reflection exercise below. Please refer to an extraction of the original article overleaf for a description of MISS-21 and an explanation of how patient satisfaction relates to patient outcomes. Now you may refer to the specific questions in the 4 domains if there are domains or items of concern.

**PTA Exercise:**

Your task: Please complete the following questions based on the feedback from the past PTA sessions and return this worksheet at your final PTA session.

1. How do you think your sessions went?
2. What went well and why?
3. What could be done better and how?
4. Summarise strengths and up to three things to focus on in my last PTA session.

My strengths: Three things to focus on:

1.

2. 3.

**About MISS-21**

Patient satisfaction is important not only as a measurement of quality of care in terms of acceptability to individual patients or populations but also as a predictor of important health outcomes. Satisfaction with consultation predicts compliance with treatment, which has both implications for the effectiveness of the medical professions’ treatments and economic implications in terms of wasted medicines. It also predicts whether patients re-attend for treatment. Clearly this may also have implications for the effectiveness of both therapeutic and preventive interventions. There is also evidence that higher patient satisfaction is associated with improved health status.

The four subscales of MISS-21 are Distress relief, communication comfort, rapport and compliance. These subscales are fairly discrete but overlapping aspects of satisfaction. They correlate with satisfaction with feeling concerns understood, explanations, relief of concerns, relationships and treatment. There were no associations between patient demographic variables or practice type and non-completion.

Please refer to the specific question(s) of concern on the MISS-21 form to proceed with the reflection exercise and come up with your plan to improve your clinical consultation. Good luck!

Reference:

Meakin R and Weinman J. The ‘Medical Interview Satisfaction Scale’ (MISS-21) adapted for British General Practice. Family Practice, 2002. Jun;19(3):257-63.
